# Contribution of Viral Mimics of Cellular Genes to KSHV Infection and Disease

**DOI:** 10.3390/v6093472

**Published:** 2014-09-19

**Authors:** Shuhei Sakakibara, Giovanna Tosato

**Affiliations:** 1 Department of Molecular Immunology, Research Institute for Microbial Diseases, Osaka University, Suita, Osaka 565-0871, Japan; E-Mail: sakakibara@ragtime.biken.osaka-u.ac.jp; 2 World Premier International Immunology Frontier Research Center, Osaka University, Suita, Osaka 565-0871, Japan; 3 Laboratory of Cellular Oncology, National Cancer Institute, National Institutes of Health, Bethesda, MD 20982, USA

**Keywords:** KSHV, vIL-6, vFLIP, NF-κB, Kaposi sarcoma, primary effusion lymphoma, multicentric Castleman disease, inflammation, tumor angiogenesis

## Abstract

Kaposi’s sarcoma-associated herpesvirus (KSHV, also named Human herpesvirus 8 HHV-8) is the cause of Kaposi sarcoma (KS), the most common malignancy in HIV-infected individuals worldwide, primary effusion lymphoma (PEL) and multicentric Castleman disease (MCD). KSHV is a double-stranded DNA virus that encodes several homologues of cellular proteins. The structural similarity between viral and host proteins explains why some viral homologues function as their host counterparts, but sometimes at unusual anatomical sites and inappropriate times. In other cases, structural modification in the viral proteins can suppress or override the function of the host homologue, contributing to KSHV-related diseases. For example, viral IL-6 (vIL-6) is sufficiently different from human IL-6 to activate gp130 signaling independent of the α subunit. As a consequence, vIL-6 can activate many cell types that are unresponsive to cellular IL-6, contributing to MCD disease manifestations. Here, we discuss the molecular biology of KSHV homologues of cellular products as conduits of virus/host interaction with a focus on identifying new strategies for therapy of KS and other KSHV-related diseases.

## 1. Introduction

Kaposi’s sarcoma-associated herpesvirus (KSHV) was identified in 1994 as a novel human herpesvirus that was shown to be the cause of Kaposi sarcoma (KS) [1]. Soon thereafter, HIV/AIDS-associated primary effusion lymphoma (PEL) and multicentric Castleman’s disease (MCD) were linked to KSHV infection [2,3,4,5]. More recently, KICS (KSHV inflammatory cytokine syndrome) was described in HIV-infected patients [6]. All or some of these diseases can co-exist in individual AIDS patients. It is now recognized that while KSHV infection is necessary, it is not sufficient for development of these malignancies. For example, the epidemiology of KSHV shows that the frequency of KSHV infection worldwide is much higher that the frequency of KS, PEL, MCD and KICS [7,8]. KSHV cannot immortalize or transform primary cells of any lineage. In KS, the KSHV-infected “spindle” cells represent a minority of cells within KS lesions within a heterogeneous mixture of other cells, including endothelial cells and inflammatory cells. These other cell components appear critical for the development and progression of KS [9,10].

The disclosure of the entire DNA sequence of KSHV prompted investigation on several viral gene homologues of cellular genes, presumably pirated by the virus during evolution [11]. Since their discovery, many studies have focused on the identification of the roles of the KSHV-pirated genes in the development of KSHV-associated diseases. In this review, we discuss biochemical and functional features of these KSHV genes and gene products, and how we can use this knowledge to target viral gene products that play multiple roles in the viral life cycle, host cell transformation and tumorigenesis.

## 2. KSHV-related diseases: KS, MCD, KICS and PEL

### 2.1. KS

The Hungarian dermatologist Moritz Kaposi first described KS in the 1970s, before HIV and KSHV were discovered. Since the worldwide spread of HIV/AIDS, KS has been recognized as one of the AIDS-related diseases. Several epidemiologic types of KS are recognized; these include classic KS (usually arising in elderly men in regions surrounding the Mediterranean Sea), endemic KS (arising in HIV-negative individuals from Africa prior to the AIDS epidemic), epidemic KS (in HIV-infected individuals) and post-transplant KS (in transplant recipients). All these KS types are the same disease.

**Figure 1:**
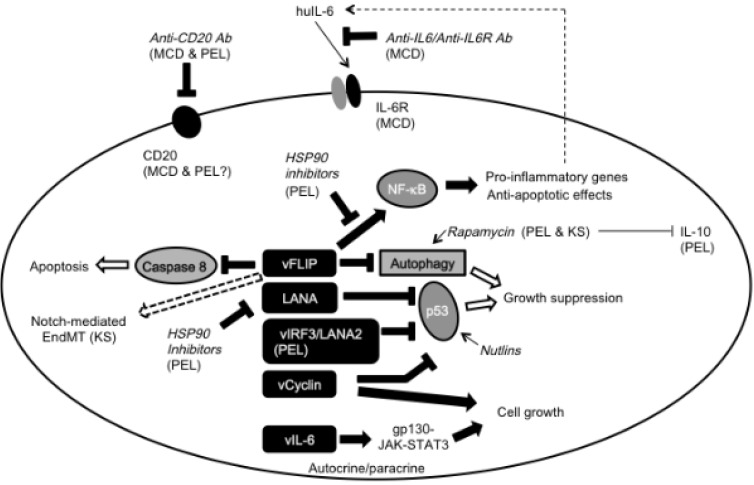
KSHV gene products and potential viral targets for the treatment of KSHV diseases. vFLIP induces pro-inflammatory genes and inhibits cell death by apoptosis and autophagy. vFLIP also enhances Notch-mediated EndMT. Several viral factors, including LANA, vIRF3/LANA2 and vCyclin inhibit p53 function and promote cell cycle progression. Autocrine/paracrine vIL-6 activates STAT3 via gp130. HSP90 inhibitors can target vFLIP and LANA, and could be effective in PEL. Rapamycin induces autophagy and inhibits vIL-10 secretion in PEL cells. Nutlins are p53 activators that could override p53 inhibition induced by several KSHV gene products. Anti-CD20 antibody has shown efficacy in some patients with MCD and PEL. Anti-IL-6R and anti-IL-6 antibodies have shown efficacy in the treatment of MCD.

KS is a multi-focal endothelial tumor with a considerable inflammatory component and vascular proliferation. The multi-focal nature of KS is not due to metastatic spread from a primary tumor but rather has been attributed to blood colonization of independently infected circulating endothelial cells/endothelial precursors [12,13,14] or opportunistic spread of KSHV [15]. The KSHV-infected tumor cells, the “KS cells” are generally not clonal and represent a minority of cells within KS lesions. Endothelial cells are almost certainly the cells of origin of KS cells [16], but their phenotype and spindle cell morphology indicates that they are not comparable to the normal endothelium. KSHV infection of vascular endothelial cells causes the spindle cell morphology, which is attributable to expression of the KSHV-vFLIP (*ORF K13*) protein [17,18,19]. The KSHV-infected KS cells express the lymphatic endothelial cell markers VEGFR3, LYVE-1, VEGF-C, and Prox1, attributable to expression of KSHV-vIL-6 protein [20,21,22]. KSHV also induces endothelial-mesenchymal transition (EnMT) characterized by reduced expression of the endothelial cell markers CD31, VE-cadherin, CD34 and Tie2, and expression of the mesenchymal markers αSMA (Acta2), NG-2 and PDGFRβ associated with increased cell motility [23,24]. This KSHV-induced transdifferentiation of endothelial cells is associated with activation of canonical Notch signaling (Figure 1), which provides a growth advantage to the KSHV-infected endothelial cells and is initiated by vFLIP (*ORF K13*) and vGPCR (*ORF74*) via incompletely defined pathways [23,24,25].

### 2.2. MCD and KICS

Multicentric Castleman disease (MCD) is a systemic lymphoproliferative disorder characterized by intermittent flares of severe inflammatory symptoms that include fever, night sweats, splenomegaly and lymphadenopathy associated with laboratory symptoms of hypoalbuminimia and anemia [26,27]. Characteristically, circulating levels of certain inflammatory cytokines, including IL-6 and IL-10, are elevated. The diagnosis of MCD is based on specific histologic features of the lesions [28]. This includes plasma cell infiltration of the mantle and inter-follicular zones of affected lymph nodes, which generates characteristic concentric layers that resemble the skin layers of onions, and increased vascularization of the interfollicular space.

With the spread of the AIDS epidemic, it was realized that MCD occurs at a higher rate in patients with HIV/AIDS and that in these patients MCD is almost universally associated with KSHV infection [4,5,29]. KSHV-LANA (latency-associated nuclear antigen)-expressing B cells, which are scattered towards the periphery of the affected follicle, are generally monotypic IgM/Igλ-expressing B cells [6,30]. vIL-6 is often detected in the circulation [6,31], particularly during disease flares, and circulating KSHV is usually present at high levels [26,32]. Recently, an MCD-related syndrome was identified and named KICS (KSHV Inflammatory Cytokine Syndrome): the clinical symptoms of KICS are indistinguishable from those in MCD, but enlarged lymph nodes are not observed and the histologic diagnosis of MCD is missing. Levels of IL-6, vIL-6 and IL-10, and KSHV viral load are comparably high in KICS and HIV-associated KSHV-MCD, and much higher than observed in KS [6,27].

KSHV gene products detected in MCD lesions include vIL-6 (*ORF K2*), PF-8 (*ORF59*), LANA (*ORF73*) and the vIRFs (*ORFs K9*, *K10/10.1*, *K10.5*, *K11* and *K11.1*), indicating that KSHV may be in its lytic phase, in at least a proportion of the infected cells [6,27,33,34,35].

There is no standard therapy for MCD. Siltuximab, a chimeric neutralizing monoclonal antibody against IL-6 has recently received FDA approval for use in HIV-negative and KSHV-negative MCD. Tocilizumab, a humanized neutralizing antibody against the IL-6R is approved in Japan as a therapy for KSHV-positive and KSHV-negative MCD. Several studies have shown that IL-6/IL-6R targeting (Figure 1) reduces MCD-associated lymph node swelling and fatigue [36,37]. Clinical benefit from the successful targeting of IL-6 or its receptor IL-6R in patients with MCD supports a contributing role of this cytokine in disease pathogenesis and symptomatogy. vIL-6, which is often measurable during MCD flares, is not usually neutralized by IL-6-neutralizing antibodies due to epitope differences [33]. Nonetheless, recent pre-clinical studies have shown that vIL-6 requires some level of IL-6/IL-6R signaling for activity, suggesting that IL-6/IL-6R targeting may also serve to reduce vIL-6 activity [38]. Yet targeting vIL-6 could be a treatment worth investigation in MCD when other treatments are ineffective. Rituximab, a humanized monoclonal antibody against the B-cell marker CD20 has shown efficacy in some cases of MCD (Figure 1) [39].

### 2.3. PEL

PEL is a rare and aggressive non-Hodgkin’s lymphoma that typically presents as a liquid malignancy in the body cavities of patients with HIV-AIDS [40]. PEL cells are always infected with KSHV and often are co-infected with EBV [2,40]. Although PEL cells are of B-cell lineage as they display immunoglobulin gene rearrangement, they express the surface markers CD45, CD38, CD71 and CD30, but lack expression of CD20, CD19, surface immunoglobulin, CD79a and other typical B cell surface markers. Despite the absence of surface CD20, there is evidence that rituximab (anti-CD20 antibody) can be an effective treatment for some patients with PEL (Figure 1) [41,42]. Morphologically, PEL cells appear plasmablastic, immunoblastic or anaplastic lymphoid cells.

KSHV does not immortalize B-lymphocytes in culture and success in adaptation of primary PEL cells to culture has been limited. However, a few PEL cell lines have been established from PEL patients, which have been critical to KSHV research as they maintain KSHV infection through passage in culture. KSHV is necessary for the survival of established PEL cell lines [43,44,45]. In PEL cells KSHV is maintained as an oligoclonal or monoclonal episome, and is mostly latent although a small proportion of cells can spontaneously undergo lytic replication associated with vIL-6 expression [46,47]. Viral replication can be induced experimentally in PEL cells with TPA treatment [48]. A characteristic feature of PEL is high level VEGF secretion; VEGF is critical to increased vascular permeability and production of body cavity effusions that is typical of PEL disease, and through these functions contributes to PEL disease progression [49,50]. Cytogenetic characterization of PEL cells has failed to detect common chromosomal aberrations, but *Myc* is generally amplified [40]. KSHV LANA, which maintains the viral genome during cell division, functionally inhibits the tumor-suppressor genes p53 and Rb [51].

## 3. KSHV-pirated Inflammatory Genes: vIL-6, vFLIP and vMIPs 

vIL-6 (*ORF* *K2*) is expressed in MCD lesions and in PEL cells in conjunction with LANA (*ORF73*) and other KSHV genes, including PF-8 (*ORF59*) and *vIRFs* (*ORFs K9*, *K10/10.1*, *K10.5*, *K11* and *K11.1*) [6,27,33,34,35]. Patients with MCD and KICS have detectable vIL-6 in the circulation, and flares of MCD are associated with spikes in circulating levels of vIL-6 [6,31].

The amino acid sequence of vIL-6 exhibits approximately 25% of similarity to that of human IL-6 [48,52]. Consistent with this modest amino acid conservation, signaling by cellular and vIL-6 differ. Cellular IL-6 requires binding to the non-signaling IL-6R prior to engagement of the signaling chain gp130 [53]. Instead, vIL-6 directly ligates and activates gp130 signaling without a requirement for IL-6R binding [33,54]. Since the distribution of gp130 is much wider than that of IL-6R, it follows that vIL-6 may affect a wider range of cells than its cellular counterpart, which requires the alpha subunit of the receptor, IL-6R. vIL-6 is inefficiently secreted. Nonetheless, vIL-6 can also signal from the intracellular compartment through direct binding to intracellular gp130 [55,56].

An early study reported that subcutaneous inoculation of vIL-6-expressing fibroblasts in nude mice resulted in accelerated fibroblast growth and formation of tumors that were much larger and more vascularized than observed in controls injected with control fibroblasts; tissue levels of VEGF were much higher than in controls [50]. vIL-6 may play a similar growth-promoting, permeability-enhancing and pro-angiogenic role in KSHV-MCD, KICS and PEL, conditions in which vIL-6 is detected in the circulation [6]. The potential importance of vIL-6 in MCD is confirmed by studies of vIL-6 transgenic mice: H2K promoter-driven vIL-6 expression in hematopoietic cells caused high mortality in most of the founder mice; in the surviving mouse lines, splenomegaly, lymph node enlargement and other manifestations typical of MCD were observed [38].

### 3.1. vFLIP

KSHV-infected cells in KS lesions, the “KS cells” show a characteristic spindle cell shape. KS cells express latency-related genes, including LANA, vFLIP (*ORF 71*) [viral Fas-associated death domain (FADD) interleukin-1β-converting enzyme (FLICE) inhibitory protein] and kaposin (*ORF K12*), and lytic genes, including vGPCR (*ORF 74*) and vCyclin (*ORF72*) [57]. Intriguingly, KSHV vFLIP alone is sufficient to change the typical cobblestone morphology of endothelial cells into that of elongated, spindle-like cells [17,18,19]. vFLIP was originally identified as a viral homologue of cFLIP (cellular FLICE-like inhibitory protein), which inhibits Caspase 8 activity induced by death domain-containing receptors [58]. vFLIP activates the canonical NF-κB pathway (Figure 1), and the morphologic change into spindle-cells induced by vFLIP is dependent upon vFLIP activation of the NF-κB pathway. Constitutive NF-κB activation leads to transcriptional regulation of NF-κB target genes, including increased expression of proinflammatory cytokines (*GM-CSF*, *IL-6* and *IL-1β*), chemokines (*Mip1α*, *Rantes*, *Mcp-2*, *Ip-10* and *I-tac*) and interferon-responsive genes, which are likely critical contributors to the prominent proinflammatory phenotype of KS [19,57]. Furthermore, persistent endothelial NF-κB activation by vFLIP induces expression of the NF-κB regulator A20/TNFAIP3, which represses vFLIP-induced NF-κB activation and augments IKK1 protein expression [59]. A20, a ubiquitin-editing enzyme, inhibits NF-κB activation by TNFα and vFLIP, albeit through distinct mechanisms [59]. When TNFα induces NF-κB activation, A20 ubiquitinates IKKγ promoting proteasome-dependent degradation and reducing downstream signaling [60]. Instead, when vFLIP-induces NF-κB activation, the de-ubiquitination activity of A20 is dispensable for NF-κB inhibition [59]. High-level expression of A20 in vFLIP-expressing cells and tissues suggests an important role of the NF-κB pathway in KS [59].

Another function of vFLIP is regulation of cell death by autophagy, a tightly regulated process of cell degradation leading to the removal of cytoplasmic cell components [61]. vFLIP inhibits autophagy and promotes cell survival. This pro-survival function is mediated by vFLIP binding to Atg3, preventing Atg3 binding to the ubiquitin-like protein LC3, which is critical for autophagosome biogenesis [61]. Rapamycin, an mTOR inhibitor with anti-tumor activity, is a potent inducer of autophagy. Despite vFLIP inhibiting Rapamycin-induced autophagy [61], Rapamycin stops growth and KSHV reactivation in PEL cells, which express vFLIP (Figure 1) [62,63,64]. In addition, Rapamycin reduces PEL and KS tumor progression in mice [63,65]. The anti-PEL activity has been attributed to Rapamycin inhibiting secretion of IL-10, an essential autocrine growth factor for PEL [62,63].

### 3.2. vMIPs

KSHV encodes the proteins vMIP-I (*ORF K6*), vMIP-II (*ORF K4*) that share extensive sequence identity (43% and 52%, respectively) to the cellular cytokine MIP1α (macrophage inflammatory protein/CCL3), and vMIPIII (ORF K4.1), which is more distantly related to MIP1α [66,67]. vMIP-1 binds to the CCR8 receptor, resulting in Ca^2+^ ion-dependent signaling [68]. vMIP-2 was shown to bind to CCR3 and CCR8 acting as an agonist and to also bind to a variety of other chemokine receptors acting as a broad spectrum antagonist [68,69,70,71]. vMIP-III preferentially binds and activates CCR4, acting as a chemoattractant for Th2-type memory T cells, which express this receptor in the skin [67,72]. The cutaneous location of KS has suggested an important role of vMIP-III as an attractant of pro-tumorigenic Th2-type cells that would favor KS progression rather than Th1-type cells, which usually exert anti-tumor activity [67]. Importantly, all three vMIPs were shown to exert pro-angiogenic activity in model systems, and it was proposed that they contribute to the pro-angiogenic phenotype of KS and MCD [67,70]. vMIPs are expressed in the lytic phase of KSHV infection, and have been detected in KS and MCD, which contain spontaneously reactivated cells: vMIP-I and vMIP-II were detected in tissues affected with MCD, whereas only vMIP-II was detected in KS tissues [73].

Currently, there are no preclinical models to assess vMIPs function *in vivo*, and the complexities of chemokine receptor/ligand interactions and redundancy of viral and cellular chemokines suggests that specific targeting these viral products may be difficult. An intriguing approach to overcome some of the complexities of the chemokine system has focused on the development of a therapeutic neutralizing antibody that inactivates multiple chemokines [74].

## 4. The NF-κB and p53 Pathways: Common Targets of KSHV Gene Products Relevant to KSHV Malignancies

Non-Hodgkin’s lymphomas generally display constitutive activation of NF-κB due to defects in its homeostatic control [75]. KSHV constitutively activates NF-κB via physical interaction of vFLIP with IKKγ/NEMO within the I-κB kinase (IKK) complex (Figure 1) [76,77]. The molecular chaperone HSP90 binds to the vFLIP/IKK complex in PEL cells (Figure 1) [78]. Consistent with an essential function of NF-κB activity in sustaining PEL cell survival, inhibition of NF-κB results in PEL cell death [79,80]. In addition, inhibition of HSP90 or vFLIP kills KSHV-infected PEL cell lines, inducing apoptosis and autophagy [44,78,81,82]. The function of HSP90 is to maintain or promote the proper conformation of other “client” proteins, which include many oncogenes and KSHV LANA [83,84,85]. Inhibition of HSP90 causes “client” proteins to acquire abnormal conformation leading to their ubiquitination and proteasome degradation [84]. Thus, HSP90 inhibitors (Figure 1) are being developed as potential therapeutics in cancer, and some drugs have reached clinical testing in different cancer types, but not in PEL or other KSHV-related malignancies [86,87]. Since HSP90 regulates the stability of several IKK kinases and disruption of HSP90 blocks NF-κB activation [88,89], HSP90 inhibitors hold promise in the treatment of PEL.

Other than vIL-6 and vFLIP, there are several viral inflammatory factors encoded by the KSHV genome [48]. vIRF1 (*ORF K9*), which aligns in the middle of viral genome [11], inhibits type-I IFN signaling and expression of genes under IFN regulatory control [90]. vIRF3/LANA2, which is expressed in PEL but not in KS tissues, inhibits p53 transcriptional activity and pro-apoptotic function (Figure 1) [91]. An important non-inflammatory viral homologue of a cellular protein is vCyclin, which can overcome retinoblastoma (RB) tumor suppressor protein-mediated cell cycle arrest [92], and inhibit p53 expression and function [93]. Transgenic expression of vCyclin in mice under the control of VEGFR3, alters lymphatic vessel structure and causes lymphatic vessel dysfunction [94]. Other than viral homologues, LANA, which is expressed in all KHSV-infected cells, also interacts with p53 and inhibits its transcriptional activity (Figure 1) [51]. The oncoprotein MDM2 (Monocyte to Macrophage Differentiation Factor-2) binds to p53 and negatively regulates its stability and pro-apoptotic activity [95]. Thus, inhibitors of MDM2 would be expected to activate p53 and increase cell death, which would be a valuable approach to reducing tumor cell growth. Nutlins are selective inhibitors of the p53-MDM2 interaction, which cause p53-dependent apoptosis in cancer cells (Figure 1) [96]. Nutlin-3a, a small molecule inhibitor of the p53/MDM2 interaction, which promotes p53 reactivation, kills PEL cells in culture and has potent anti-tumor activity in mice bearing PEL tumors [97,98].

## 5. Future Directions

We have described KSHV-pirated genes and their functions in KSHV-related diseases. It is clear that viral homologues of cellular genes that have been captured during virus-host co-evolution play important roles in KSHV life cycle and pathogenicity. Therefore, a clear understanding of the function of these factors can provide precise therapeutic targets directed at the pathogen. Precision medicine is a current goal in cancer therapy, as it ideally targets important unique tumor targets while sparing the normal cell counterparts. Targeting KSHV-specific factors is in line with current efforts.
